# Biogenic Amines in Meat and Meat Products: A Review of the Science and Future Perspectives

**DOI:** 10.3390/foods11060788

**Published:** 2022-03-09

**Authors:** Maria Schirone, Luigi Esposito, Federica D’Onofrio, Pierina Visciano, Maria Martuscelli, Dino Mastrocola, Antonello Paparella

**Affiliations:** Faculty of Bioscience and Technology for Food, Agriculture and Environment, University of Teramo, Via R. Balzarini, 1, 64100 Teramo, Italy; mschirone@unite.it (M.S.); lesposito2@unite.it (L.E.); fdonofrio@unite.it (F.D.); dmastrocola@unite.it (D.M.); apaparella@unite.it (A.P.)

**Keywords:** biogenic amines, meat, meat processing, tyramine, histamine, shelf-life

## Abstract

Biogenic amines (BAs) can be found in a wide range of meat and meat products, where they are important as an index for product stability and quality, but also for their impact on public health. This review analyzes the scientific evidence gathered so far on the presence and role of biogenic amines in meat and meat products, also considering the effect of technological conditions on BAs accumulation or decrease. The data provided can be useful for developing solutions to control BAs formation during the shelf-life, for example by novel starters for dry cured products, as well as by packaging technologies and materials for fresh meats. Further research, whose trends are reviewed in this paper, will fill the knowledge gaps, and allow us to protect such perishable products along the distribution chain and in the home environment.

## 1. Introduction

Biogenic amines (BAs) are low molecular weight compounds with biological activity, produced by the decarboxylation of amino acids or amination and transamination of aldehydes and ketones during the metabolic processes in living cells [1]. They can be normally present in plants, animals, and humans, where they exhibit essential physiological functions, such as neurotransmission, regulation of growth and blood pressure, and other important roles in the intestinal immune system [2]. However, when great amounts are introduced through the consumption of contaminated foods and/or beverages, they can cause adverse effects on nervous, respiratory, and cardiovascular systems and/or allergic reactions, especially in individuals with mono- and diamino-oxidase deficiency, or assuming drugs that inhibit such enzymes [3]. The main symptoms of histamine intoxication, occurring within few hours, are nausea, vomiting, diarrhea, headache, urticaria, tachycardia, and even death, while tyramine poisoning is characterized by headache, palpitations, nausea and vomiting, and a rise in blood pressure [4,5,6].

BAs are also considered an index of food spoilage, as high concentrations can be found when the hygiene quality of the product decreases [7]. The most common BAs occurring during the deterioration of foods are cadaverine (CAD), putrescine (PUT), spermidine (SPD), spermine (SPM), β-phenylethylamine (PHE), tyramine (TYR), and histamine (HIS) [8]. However, SPD and SPM are also naturally occurring BAs in fresh meat [4]. 

**Figure 1:**
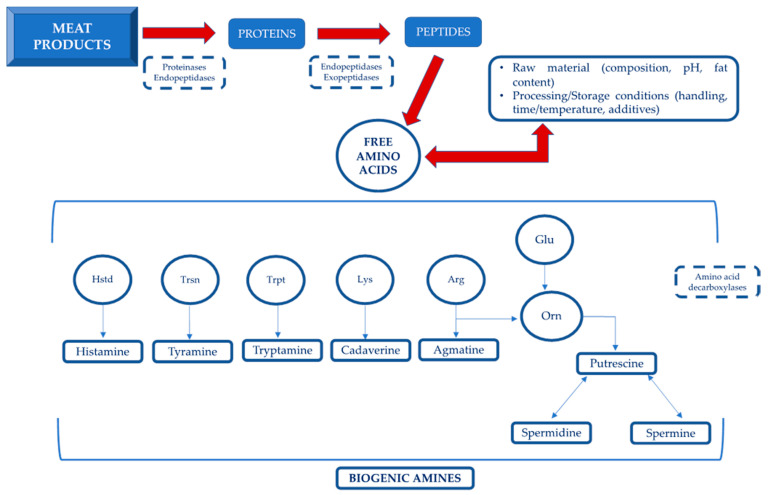
Scheme of biogenic amines formation. Legend: Hstd = Histidine; Trsn = Tyrosine; Trpt = Tryptophan; Lys = Lysine; Arg = Arginine; Glu = Glutamine; Orn = Ornithine.

Several factors associated with raw materials, such as pH and chemical composition, as well as some handling and manufacturing operations (e.g., fermentation, ripening or post contamination) but also temperature and time of storage, may influence their presence in foods [9]. The corresponding precursor amino acids and the main characteristics contributing to BAs formation are shown in Figure 1. The decarboxylase-positive microorganisms can be present in raw materials and/or introduced by contamination before, during or after processing [10]. Many genera (i.e., *Escherichia*, *Klebsiella*, *Citrobacter*, *Proteus*, *Shigella*, and *Salmonella*) belonging to the *Enterobacteriaceae* family, as well as some *Micrococcaceae* (*Staphylococcus* and *Micrococcus* genera) can be involved in BA production Moreover, some strains of lactic acid bacteria (LAB) of genera *Enterococcus*, *Lactobacillus*, *Carnobacterium*, *Pediococcus*, *Lactococcus*, and *Leuconostoc* can decarboxylate amino acids [11].

Meat and meat products are particularly subjected to BAs production due to their high protein and amino acid content, and the proteolytic activity can arise as a consequence of a prolonged storage or in association with the production process. The first BAs naturally occurring in fresh meat are SPM and SPD, which can be found at levels between 20 and 60 mg/kg and about 10 mg/kg, respectively [12]. During storage, the amounts of other BAs (i.e., HIS, CAD, PUT, and TYR) can also increase by the proteolysis of proteins to large peptides, which are then degraded to oligopeptides and free amino acids [4]. A significant CAD and PUT increase (>15 mg/kg) observed in raw pork meat during storage has been considered index of spoilage [13]. In Table 1, some studies from literature about concentrations of major BAs in different meat and meat products were reported.

Proteolysis is favored by intrinsic factors, such as acidity increase, dehydration, and the action of sodium chloride in some meat derived products, but also by microbial activity during fermentation and/or other food production processes [14]. Although meat fermentation promotes preservation against various pathogenic and spoilage microorganisms, BAs accumulation in fermented meat products has also been reported [15]. Due to the importance of their adverse health effects and specific concerns in food hygiene, BAs individually or in combined forms can be used as important indicators of freshness, quality, and spoilage in meat and meat products [12]. Firstly, a Biogenic Amine Index (BAI) calculated from the sum (mg/kg) of PUT, CAD, TYR, and HIS, was proposed for cooked meat products allowing a four-scale classification. If BAI value is less than 5 mg/kg, the meat is fresh and of good quality, between 5 and 20 mg/kg it is still acceptable with some signs of deterioration, between 20 and 50 mg/kg and above 50 mg/kg the meat is of low quality and scarce hygiene quality (spoiled), respectively [16]. Moreover, the ratio between SPD and SPM was used as an index for the evaluation of chicken meat quality [17], as well as the levels of CAD and TYR were proposed to control beef and poultry spoilage during storage [18]. However, the effectiveness of BA indicators can change based on many factors, such as handling, salting, canning, modified atmosphere and so forth, and therefore, they can be more suitable in fresh than fermented or heat-treated meat products [19].

It is well known that fermented meat products generally show the highest BAs concentrations, and TYR is the most represented amine in cured meat products. However, according to the European Legislation (Commission Regulation EC No 2073/2005 and further amendments), maximum limits have been established only for HIS in fish and fish products, specifically in fish with high free histidine content in the muscle tissue, while no standards or guidelines are reported for meat products. With regards to the export of these products to Third Countries, specific agreements are set between the competent authorities of European Union (EU) Member States and the nations where they are destined. Meat and meat products can only enter in EU if they have been produced from raw materials obtained in establishments compliant with the requirements referred to Regulation (EC) No 853/2004, or with requirements recognized to be at least equivalent thereto.

This review will deal with BAs occurrence in raw meat and meat products produced by several manufacturing processes, whose role in BAs formation is discussed, taking into account both food quality and safety aspects, as well as the trend in scientific research.

**Table 1:** Biogenic amines levels in different meat and meat products reported in some studies from the literature.

Category		Biogenic Amines (mg/kg)								Reference
		PUT	CAD	HIS	SPD	SPM	TYR	PHE	TRYP	
Raw meat	Beef	6.6–90.9	<0.5–295.6	<0.5			11.1–65.5			Rosinská and Lehotay, 2014 [20]
	Pork	12.7–131.5	13.6–440.2	<0.5			34.4–55.2			
	Poultry	<0.5–382.7	<0.5–764.2	<0.5–180.5			<0.5–171.2			
	Beef	tr-1.9	nd-1.9	0.3–1.8	0.2–4.1	0.2–3.9	0.1–0.5			Jasim and Sdkhan, 2015 [21]
	Beef liver	1.5–26.1	nd-42.1	tr-136.7	5.0–10.4		4.6–12.5			Eldaly et al., 2016 [22]
	Different kinds of meat *	nq-124.0	nq-124.0	nq-55.0	nq-229.0	nq-261.0	nq-199.0			Molognoni et al., 2018 [23]
	Pork leg	0.6–14.6	nd-16.2	nd	2.6–3.9	25.2–27.6	0.7–16.6	nd-1.7	nd-6.6	Triki et al., 2018 [12]
	Lamb leg	1.2–10.1	nd-5.1	nd	8.1–12.0	31.4–40.9	0.1–10.7	0.8–9.1	nd	
	Turkey leg	1.2–68.7	nd-13.3	nd	7.3–18.3	32.6–49.2	nd-6.9	0.2–15.1	nd	
	Chicken breast	1.2–52.0	nd-14.3	0.5–2.1	6.2–9.8	41.9–53.6	nd-35.2	nd-16.9	0.4–15.8	
	Beef leg	1.3–7.4	nd	nd-0.5	2.3–5.4	25.1–33.0	0.3–1.6	0.5–2.6	nd	
	Camel and offals	0.4–0.8	0.2–0.7	nd-0.3		0.1–0.5	0.2–0.6			Tang et al., 2019 [24]
	Chicken breast muscle	1.0–1.8	<LOQ-10.5	1.4–4.3			<LOQ-4.2			Wojnowski et al., 2019 [25]
	Imported meat **	1.2–3.0	nd-4.3	0.6–1.4	1.6–6.3	2.6–11.1	0.3–2.0	nd-0.1	tr	Algahtani et al., 2020 [26]
	Broiler chicken (breast and thigh)	tr	tr	0.1–0.4	tr-0.6	tr				Saewan et al., 2021 [27]
	Minced beef	4.0–60.1	26.0–116.2	27.2–90.1			nd-60.4			Mahmoud et al., 2021 [28]
	Pork belly	0.6–63.3	0–98.3	0–1.5	3.0–3.5		5.2–76.7			Cho et al., 2021 [29]
	Pork belly, marinated	0.4–21.1	0–58.3	0–1.1	3.1–3.5		5.2–68.1			
Processed meat products	Fermented sausages	0–505.0	0–690.0	0–515.0			0–510.0			Papavergou et al., 2012 [30]
	Dry fermented meat	nd-225.1	nd-16.8	nd-151.8			nd-228.1	nd-42.7		Buňka et al., 2012 [31]
	Greek sausages	0–491.7	0–1014.1	0–375.8	1.5–19.5	13.4–60.1	3.7–381.4	0–56.4	0–60.5	Papavergou, 2011 [32]
	North European sausages	0.4–229.0	nd-246.8	nd-131.0	1.0–6.6	nd-12.0	1.3–302.9	nd-54.4	nd-109.7	De Mey et al., 2014 [14]
	South European sausages	0.3–316.4	nd-641.4	nd-131.0	nd-13.3	nd-21.1	nd-410.8	nd-57.1	nd-109.7	
	Fermented sausages	nd-564.5	9.9–654.7	nd-177.4		100.6–328.6	19.2–502.8	nd-4.3	nd-32.8	Xie et al., 2015 [33]
	Fermented beef sausages	1.0–15.8	0.5–9.0	0.3–19.6	nd-103.3	96.4–364.1	64.1–275.1	nd-16.1	nd-32.8	Çiçek, 2016 [34]
	Chinese Sichuan-style sausages		19.1–376.5	114.0–327.4			88.8–285.9			Sun et al., 2016 [35]
	Portuguese sausages	11.6–265.4	nd-364.8	nd-28.9	nd-11.5	nd-41.0	nd-150.3	nd-38.6	nd-67.1	Laranjo et al., 2017 [36]
	Belgian sausages	0.3–316.0	0–641.0	0–131.0			0–411.0			Lorenzo et al., 2017 [37]
	Turkish style sausages	1.0–24.6	72.2–320.0	5.2–99.9	34.4–68.7	2.7–20.0	69.4–162.4	2.3–7.6	20.0–40.9	Ekici and Omer, 2018 [38]
	Dry-fermented sausages	nd-212.0	nd-30.8	nd-9.7			nd-147.0	nd-36.0	nd	Ikonic et al., 2019 [39]
	Chinese sausages	nd-277.1	nd-670.9	nd-209.6	2.4–23.5	7.5–36.5	nd-209.6	nd-8.2	nd-22.5	Li et al., 2019 [40]
	Brazilian commercial salamis	91.5–818.5	37.9–166.4	nd-500.2	51.2–55.8	96.7–151.9	91.3–346.9	nd-375.9	nd-123.9	Roselino et al., 2020 [41]
	Italian commercial salamis	nd-381.2	nd-215.9	nd-240.9	nd-99.7	102.8–141.2	nd-270.0	nd-316.4	nd-297.1	
	Mortadella di Campotosto	nd-186.8	nd-15.0	nd-17.0	40.4–79.4		51.3–235.9			Serio et al., 2020 [42]

Legend: PUT = Putrescine; CAD = Cadaverine; HIS = Histamine; SPD = Spermidine; SPM = Spermine; TYR = Tyramine; PHE = Phenylethylamine; TRYP = Tryptamine; nd = not detected; tr = traces; nq = not quantified; * = cooked sausages, mortadella, cooked ham, bacon, corned beef, beef jerky, canned/pouch roast/shredded beef, salami and raw sausages; ** = luncheon, hot dog, corned beef and minced meat.

## 2. Trends in Scientific Literature on Biogenic Amines in Meat and Meat Products

The finding of BAs in meat dates back to 1887, when Nencki discovered amylamine in putrefying meat [43]. Early studies postulated that BAs, in particular HIS, were formed by autolysis [44], but later it was demonstrated that BAs are mostly produced by microbial activities [45]. In the seventies of the last century, scientific research on BAs in foods was mainly focused on the detection of HIS in fish muscles [46], while in the eighties the first reports of BAs in meat products aimed at detecting different BAs and BAs-producing microorganisms during manufacturing of dry fermented salamis [47]. In the following decade, the scientific literature on BAs in meat mainly focused on fermented products, expanding, and reaching a global dimension. Some researchers evaluated the presence of BAs in different typical products [48,49], while other studies investigated the relationship between BAs and the microbial ecology of fermented meat products [50]. At the same time, the presence of BAs in fresh meat was correlated with packaging conditions [51].

At the turn of the millennium, the attention shifted onto the role of starters in the balance of BAs in fermented meats. These studies can be considered a turning point because particular attention was paid to the selection of starters capable of reducing the content of BAs of health interest, for example by exploiting amino oxidase activity of specific microorganisms, as well as by combining starters with proteolytic strains to increase the free amino acid availability and the non-protein nitrogen [52]. In the new millennium, research was focused on the effect of processing conditions, packaging, and novel technologies on BAs content in meat and meat products, as it has been recently reviewed by Paparella and Tofalo [53]. The goals achieved by these studies will be discussed in the following paragraphs.

Based on the scientific targets documented so far in the scientific literature, it is possible to predict future trends in the research on BAs in meats. Almost certainly, the great shift in the formulation of packaging materials, imposed in Europe by the so-called Green Deal approach, will impact research on new materials capable of detecting or reducing the BAs content in foods. In this respect, some papers have already been published. For example, Sirocchi et al. [54] developed a novel active packaging containing 4% *Rosmarinus officinalis* essential oil, which sharply decreased PUT, CAD and HIS, and the corresponding microbial producers in fresh meat at 4 °C. Miller et al. [55] have recently reviewed the possible applications of BA detection systems in the packaging industry.

Among the possible technologies that might be used to decrease the BAs content in meats, ozone was studied by Mercogliano et al. [56]. These authors combined prewashing of poultry carcasses with ozonized water and gaseous ozone delivery during chilled storage for 60 min every 4 h, and obtained a sharp decrease of PUT and CAD, and a shelf-life extension of 6 days.

Other possible developments of research will be in the area of microbial cultures for meat and meat products. Considering the increasing interest for the manufacturing of nitrite-free meat products, the results obtained recently by Li et al. [57] can be considered promising. These authors selected four candidates, which did not possess the BAs formation encoding genes, to use as starter cultures to degrade total BAs and nitrite.

Finally, a strategic area of research will probably be the development of sensors for BAs assessment in meat, to evaluate meat freshness and shelf-life. In this respect, Biesuz and Magnaghi [58] recently analyzed the state of the art, discussed the challenge of the BAs volatility, and developed a set of five sensors that were able to describe the entire spoilage process of chicken samples.

## 3. Biogenic Amines as Markers of Freshness and Safety in Raw Meats

Meat ensures a complete set of amino acids, energy, lipids, and micronutrients at more competitive prices than other sources [59]. Overall, the estimated increase of human population to ~10 billion in 2050 [60] confirms the urgency of a more sustainable meat market. In this scenario, as for any food chain, it is crucial to ensure food safety from both the human and animal health side, in line with the so called One Health approach [61]. 

Red meats were classified as “probably carcinogenic to humans”, and processed meats as “carcinogenic”, but they are also in charge of non-transmissible diseases as overweight and obesity [62]; at any rate, many of the compounds responsible for some of the described conditions, are coming from cooking and/or transformation processes [63]. Of course, a non-balanced intake of dense energy foods plays a distinct role in inflammation processes [64]. In a recent study, Flores et al. [65] described how for different transformed meat products, specific chemical and biological hazards can arise. For example, polycyclic aromatic amines can derive from smoking, and Maillard reaction products from cooking [63]. Among the possible hazards in meat and meat products, BAs are considered relatively frequent [66,67]. Red and white meats are considered good potential sources of BAs for several reasons. In fact, the great amounts of proteins available is an important start point for BAs development, considering that BAs mainly derive from amino acids decarboxylation. Dabadé et al. [9] compared amino acids concentration at the purchasing day vs. the production of BAs in different food categories at the end of their shelf-life. They found higher values for specific BAs such as PHE and HIS with their precursor amino acids. They also noticed a higher incidence of TYR production in foods derived from animals than in the ones derived from vegetables.

In raw meat, the presence of Bas depends on different factors, in particular meat origin, storage conditions, the specific microbiota, and meat shelf-life. Despite Bas ubiquity, their production is influenced by microbial activity and food history. In general, wrong hygiene and storage conditions can increase the incidence of Bas [68]. Among the environmental factors, temperature management is responsible both for creating the best conditions for mesophilic bacterial growth and boosting chemical/biochemical pathways for proteins catabolism. Moreover, pH is of crucial importance on Bas production. In fact, as revised by Jairath et al. [69], the pH balance can on one hand limit microbial activity for the improved acidity, and on the other hand, increase decarboxylase enzyme production from microorganisms that use it as a defense against the acidic environment. Thus, these factors are all inputs giving Bas as main output [19].

Alessandroni et al. [70] highlighted that poultry, and in particular chicken, is more susceptible to Bas accumulation, due to the specific protein composition and a softer texture in comparison with pork and beef. Triki et al. [12] evaluated different cuts of meat over time. Beef, lamb, and pork meat demonstrated a slower qualitative decay caused by Bas compared to chicken meat, characterized by the highest level of free amino acids. Despite the prominent role of the intrinsic factors, packaging solutions may control Bas increase in raw meat and meat preparations. In fact, Chmiel et al. [71] found that high O_2_ MAP better preserved chicken breast fillets from the accumulation of Bas (mainly PUT and CAD), in comparison with air and vacuum-packed breast fillets. The same study pointed out the importance of storage conditions since product stability was higher in cold rooms and dry conditions, than in display cases exposed to light. The prevalence of specific microbial groups has also been found to enhance Bas production. Li et al. [72] studied Bas evolution in air packed beef and found a decrease of polyamines as SPM over time and a slight increase of CAD and PUT. Similarly, in frozen beef cuts stored at −18 °C for a long time, an increase of PUT and CAD, more significant for the latter, indicated qualitative decay and putrefaction [73]. Li et al. [74] found it useful to use CAD and PUT to evaluate qualitative decay in pork packed under vacuum and MAP, while using TYR was more relevant for the same meat kept in pallet packaging. In general, meat quality also depends on its handling, and BAs can reveal details on incorrect management. As a matter of facts, the major cause for meat rejection from emergent economies of Africa, Asia, and Latin America, is bruising which is a superficial discoloration due to hemorrhage under the skin. BAs profiles of bruised meats were investigated along ageing, and results showed significant differences among packaging systems and between bruised and non-bruised meat, with higher BAs concentrations in bruised meats [75].

Regarding the functions of BAs in raw meat, it has to be noted that raw meats mostly contain polyamines PUT, SPD, and SPM, in particular the last two. These are also known as constitutive amines because they are involved in many bodily functions having a fundamental role in cellular communication and proliferation [76]. Due to their direct role in the latter, polyamines have also been associated to tumors development [77]. However, the same amines are recognized as potent antioxidants [78], capable of limiting DNA damages [79].

Considering the great availability of polyamines in raw meat, many researchers proposed the use of a ratio between SPM and SPD for raw meat quality evaluation [80]. The main reason for the use of this index is because it is independent from microbiological activity and relatively stable over time. In this respect, many studies agreed on using the sum CAD + PUT to evaluate raw meat decay independently from the animal species. HIS and TYR start to increase after some days of storage unless the initial microbial load is high. Sørensen et al. [81] explained the importance and the novelty in using BAs levels for screening the quality of meat products in the circular economy. Others as Beckith et al. [82] gave a wide description of BAs, total volatile nitrogen and trimethylamine value as quality indexes in muscle foods. More recently, Shashank et al. [83] underlined the need for faster methods for the assessment of meat products’ safety by means of BAs levels.

All these studies clearly show how BAs evolution in raw meat is a suitable and helpful tool for monitoring meat safety and predict meat quality. In this regard, Wojnowski et al. [25] developed a novel liquid-liquid microextraction gas chromatography–mass spectrometry method and analyzed environmental aspects but also feasibility and rapidity of the method. Based on this study, the analytical method and in particular the quantification of TRYP, CAD, PHE and PUT is considered useful for early determination of meat spoilage in poultry, pork and beef samples. Additionally, considering the strict link between BAs production and the microbiota, BAs can somehow be employed as an indirect index of microbial spoilage. Moreover, when it comes to formulated or ready to eat meat products, BAs occurrence can help in identifying both meat freshness, contaminated ingredients, and wrong hygiene practices. In this regard, the use of BAs indexes to monitor the freshness of minced meat [28], imported meat and meat products [37], ready to eat street-food [84] is documented in the scientific literature. Anyway, as reviewed by Biesuz and Magnaghi [58], there are still misleading concepts in developing newer BAs determination methods. As highlighted by Wakas et al. [85], several limitations on quantification are posed from matrix effects and sample pretreatment procedures. One of the issues limiting BAs indexes success in raw meats lies in the complexity of the matrix, where lipids, proteins and other compounds can make pretreatment procedures harder.

## 4. Biogenic Amines in Processed Meats

BAs can be found in a wide range of meat products, as documented by scientific literature [69]. Generally, fermentation and BAs production is a bond that gives results in meat or animal-based products but also in foods of vegetable origin [86]. For years, research on BAs in foods focused mostly on fermented and cured products, in particular meat products [87,88,89], considered as possible reservoirs of nitrosamines, showing carcinogenicity from processed meats intake [90,91].

More than cured or formulated products, fermented meat products represent a primary source of BAs. Due to their manufacturing process, they possess all the characteristics that favor BAs production, mainly due to the bacterial and fungal activities. In fact, microorganisms take advantage of the available nutrients, the favorable water activity (at the beginning of the process), and the anaerobic conditions ensured by the environment. The total or partial O_2_ absence can promote the specific production of amines as HIS while reducing the amounts of PUT and CAD, directly acting on specific bacteria as *Enterobacter cloacae* and *Klebsiella pneumoniae* [92]. In this rich and selective medium, bacterial growth is also promoted by the physical state of fermented products, commonly made of ground meat added of variable percentages of minced fat (according to the recipe) that ensures protection to bacterial cells. During manufacturing, pH plays a leading role, promoting decarboxylase activity. In these products, where mainly LAB cultures are employed to carry on fermentation, acidification can limit or stress BAs production [93]. Another factor that diminishes BAs formation by microorganisms is the presence of salt. Some authors [4] reported that values of 2.5–3.0 g/kg are enough to reduce HIS production. However, other authors formulated the hypothesis of a possible enhancing effect of NaCl on the metabolic activities of decarboxylating microorganisms; in particular, they supposed the essential role of Na^+^ ion in the sodium/proton antiport system, through which H^+^ ions are removed from the cell [52,94]. Furthermore, it was observed that low containing sugars environments increase decarboxylase pathways boosting BAs production [95].

In fermented meat products, different microbial groups, intentionally added (starter cultures) or part of the natural microbiome [96], can produce BAs. The meat environment is commonly characterized by *Enterobacteriaceae*, lactic acid bacteria (LAB), *Brochothrix thermosphacta*, pseudomonads and some clostridia. Obviously, storage conditions and the gaseous composition of the environment have a direct influence on the final ecology, enhancing specific groups [71]. Main bacterial groups responsible for BAs formation in meat fermented products are *Enterobacteriaceae* and pseudomonads, some strains belonging to the genera *Staphylococcus* and *Bacillus*, and LAB [57]. Fermented products as salamis and sausages can pose health hazards for the accumulation of TYR, HIS, PUT and CAD mainly. TYR was the most frequent and abundant bioactive amine found in dry fermented sausages of the Spanish retail [97]; similar results had been obtained in a study on salamis from Southern Italy [98]. TYR and HIS are linked with direct side effect on human body, but the presence of other amines can even boost their activity [99,100]. Many studies have confirmed the natural occurrence of these amines in samples coming from all over the world [38,101,102] and produced at industrial level [38]. Alves et al. [103], analyzing dry fermented sausages and salamis from Portugal and Serbia, found low amounts of TYR, CAD and PHE, while HIS was present only in two samples. Low levels of BAs usually indicate good hygiene and handling procedures, high quality of the raw materials and suitable drying/fermenting conditions.

A growing part of scientific literature analyzes how specific bacterial consortia can limit or degrade BAs formation [104]. Alvarez and Arribas [105] classified many BAs-degrading bacteria and concluded that amino oxidase activity of some strains of LAB is a criterion to select BAs-degrading bacterial cultures, although same strains can also show decarboxylase activity and therefore form BAs. Thus, bacteria can have a two-side effect on BAs final content that depends mainly on strains characteristics. As for the other features that potentially affect BAs formation, bacteriocins as nisin were tested on fish meat without any direct effect on BAs reduction [106]. Serio et al. [42] investigated the effect of casings on dry fermented salami, observing significant effects on BAs accumulation among different casings. The major expression of TYR and PUT degrading activity at the end of ripening can be caused by some strains of *Lactobacillus*, *Pediococcus*, *Micrococcus*, as well as *Staphylococcus carnosus*. Apart for the direct toxicologic effects that BAs can provoke, in the last years concerns about their involvement into N-nitroso compounds production are coming up. These toxic compounds are formed from nitrogenous compounds deriving from bacterial fermentation and proteolysis. Among them, nitrosamines are recognized as carcinogenic and highly responsible for gastrointestinal tumors [107]. Considering the widespread use of preservatives as sodium nitrites (E-250, E-251) in meat products, BAs are the main substrate for N-nitroso compounds formation. In this respect, Drabik-Markiewicz et al. [108] found that higher levels of SPD and PUT increased the quantity of N-nitrosodimethylamine, while SPD and CAD amplified the final level of N-nitrosopiperidine in heated cured pork meat. The same study evidenced the effect of rising temperatures and percentage of sodium nitrites, which were positively correlated with the final content of N-nitroso compounds. Long Yan Fong et al. [109] gave an interesting view of the development of tumors like nasopharyngeal carcinoma (also known as a Canton tumor), in correlation with the dietary styles of the lower income population from that area. The linkage regards the common habit of these consumers heating higher quantities of fermented products since childhood. Sausages and typical Chinese hams contain high levels of both proteins and BAs, in charge of the accumulation of N-nitroso compounds. In a previous study, Martuscelli et al. [66] found that different smoking processes reduce the quantity of free amino acids in dry cured hams, impacting on the final level of BAs. In this study, non-smoked hams had higher levels of BAs than smoked products. Therefore, the diffusive phenomena of salt and water being correlated to the amin acid decarboxylase and/or amino oxidase activities [9], the control of the evolution of dehydration processes could be a useful tool to contrast the accumulation of BAs in ripened meat products [110,111].

Heat treatments do not affect the BAs concentration in meat products, although they contrast microbial spoilage. Thus, the occurrence of BAs in cooked meats indicates an incorrect handling before and after product preparation. Recently, the effects of various cooking methods (boiling, grilling, microwave, and sous-vide cooking) on BAs content were investigated in different foods; Muñoz-Esparza et al. [112] demonstrated that chicken meat samples did not show any polyamine loss after the different heat treatments, while only a modest reduction of BAs was observed in beef and pork meat after cooking.

Another aspect of great importance is the assessment of the risk for infants of BAs intake with the consumption of ready-to-eat meat baby products. For the first time, Polish researchers presented a study on the amine-related risk assessment for baby foods, including meat-based formulations [113]. No significant difference (*p* < 0.05) was found in the BAs profile among the different meat species (beef, chicken, lamb, veal, rabbit, turkey, and pork). In relation to baby food products containing beef, it was recommended to reduce their consumption by infants under 12 months of age, due to the occurrence of HIS at a high level (400 ng/g).

## 5. Conclusions

BAs are present at various concentrations in meat and meat products. Despite their significance for both public health and meat shelf-life, there is a lack of knowledge on levels that could be used as guidelines in the different products that are available on the market. To date, the only BA that is regulated, at least in the European Union, is HIS, but only for fish and fish products. Since the International Agency for Research on Cancer (IARC) classified red meat as probably carcinogenic to humans, also due to the risk of nitrosamine formation, the research on BAs in meat and meat products has become increasingly important.

In the last twenty years, the scientific literature has provided evidence of different solutions that could be applied to reduce BAs formation in meat and meat products. Packaging, starters, and decontamination by means of different technologies have already proved to be useful, but much more needs to be done to protect the products along the distribution chain, and possibly in households. This goal might be achieved by investing in research on sensors and packaging, to offer cheap and easy-to-use devices for rapid BAs assessment in meat and meat products.

## Data Availability

Data is contained within the article.
